# Selective expression of HSP27 in cortex and hippocampus offers insights of TDP‐43 and APOE interaction

**DOI:** 10.1002/alz.086931

**Published:** 2025-01-03

**Authors:** Muhammad I Abeer, Michael A. Gitcho

**Affiliations:** ^1^ Delaware State University, Dover, DE USA; ^2^ Delaware Center for Neuroscience Research, Dover, DE USA

## Abstract

**Background:**

Aggregation of transactive response DNA binding protein 43 (TDP‐43) is the major pathological feature of amyotrophic lateral sclerosis (ALS) and frontotemporal dementia (FTD). Recently, in up to 50% of Alzheimer’s disease (AD) cases TDP‐43 pathology was discovered and this pathology has been referred to as limbic‐predominant age‐related TDP43 encephalopathy (LATE). Several studies reported that TDP‐43 binds to heat shock protein family B (small) member 1 (HSPB1 or HSP27) but no functional evaluation of this interaction has been explored. Inducing expression of HSP27 has been shown to be protective of many other disease conditions and has been shown to reduce aggregation of amyloid in AD. In general, the goal is to utilize both primary neuronal cultures and mice that are selectively expressing pathogenic TDP‐43, HSP27, and apolipoprotein E (APOE) in the brain to characterize the effect of HSP27 overexpression on TDP‐43 and APOE. This will give us a better model to understand TDP‐43 proteinopathies. In the present study, we hypothesize that increased expression of HSP27 may reduce TDP‐43 aggregation and alter mitochondrial morphology.

**Method:**

A new transgenic mouse model was developed to selectively drive human HSP27 and pathological TDP‐43 with a defective nuclear localization signal (DNLS) in the hippocampus and neocortex using the Ca^2+^/calmodulin protein kinase (Camk2a) tetracycline inducible system. The following genotypes have been evaluated for immunohistochemistry, biochemistry (solubility fractionation), and Western blot: wild‐type, Camk2a/DNLS, Camk2a/HSP27 and Camk2a/HSP27/TDP43DNLS at 4 months of age.

**Result:**

Preliminary *in vitro* results show that cells overexpressing HSP27 reduce aggregation and protein levels of TDP43. However, mice overexpressing HSP27 in a TDP43DNLS background in the hippocampus and cortex does not show any reduction in the soluble fraction. Interestingly, increased expression of HSP27 in the hippocampus of Camk2a/HSP27 mice showed a significant reduction of endogenous APOE expression. Immunohistochemical and bioenergetic experiments are currently being carried out to evaluate the brain and mitochondrial morphology upon HSP27 overexpression.

**Conclusion:**

Overall, our initial data suggests that modifying HSP27 expression modulates endogenous APOE level.

